# Association of Vitamin D Level in Hyperreactive Airway Diseases: A Case-Control Study

**DOI:** 10.7759/cureus.77037

**Published:** 2025-01-06

**Authors:** Dhiman M Vaghela, Namrata K Makwana, Akshay Aravind, Dhaneshwar Kumar Mandal Shankar

**Affiliations:** 1 Department of Pediatrics, Shri Meghji Pethraj Shah Government Medical College, Jamnagar, IND

**Keywords:** children, hyperreactive airway disease, nutritional status, sun exposure, vitamin d

## Abstract

Background: Low vitamin D levels have been associated with hyperreactive airway diseases (HRADs) such as wheeze-associated lower respiratory tract infections, asthma, and allergic rhinitis in children. Vitamin D insufficiency is a modifiable risk factor for managing recurrent respiratory tract infections. This study aimed to estimate vitamin D levels in HRAD patients and assess their association with HRAD by comparing them to healthy children.

Methods: This case-control study was conducted over 18 months in the Department of Paediatrics, Guru Gobindsingh Government Hospital, Jamnagar. A total of 200 children (100 cases with HRADs and 100 healthy controls) aged six months to 12 years were enrolled using simple random sampling. Cases included children with ≥2 episodes of wheeze-associated lower respiratory tract infections or asthma. Controls were healthy children who were not taking vitamin D3 supplements attending the immunization clinic. Vitamin D levels were measured using electrochemiluminescence immunoassay (ECLIA). Data on dietary habits, sun exposure, and nutritional status were collected. Chest and wrist X-rays were evaluated.

Results: The mean vitamin D level in cases (30.2 ± 17.8 ng/mL) was lower than that of controls (33.6 ± 14.5 ng/mL), with significantly higher odds of deficiency in cases (OR 3.31, p = 0.001). Vitamin D deficiency was associated with younger age, poor nutritional status, limited sun exposure, and low dietary intake of vitamin D-rich foods. Chest X-ray hyperinflation and wrist X-ray findings were significantly linked to vitamin D deficiency.

Conclusions: Vitamin D deficiency is significantly associated with HRADs in children. Screening and prophylaxis for vitamin D deficiency, particularly in at-risk groups, may help manage HRADs effectively.

## Introduction

The rising global prevalence of hyperreactive airway diseases (HRADs) like asthma and allergic rhinitis has become a major public health concern, particularly in pediatric populations [1]. Asthma alone affects over 300 million individuals worldwide, with increasing incidence in children [2]. Given the chronic and inflammatory nature of these diseases, we need to identify modifiable factors that can influence their development and severity. Among these, vitamin D3 has potential immunomodulatory effects that extend its traditional role in bone health.

Vitamin D3 influences the immune system by modulating innate and adaptive immunity, particularly through the regulation of T-helper (Th) cells, macrophages, and dendritic cells. It reduces airway inflammation by inhibiting pro-inflammatory cytokines like IL-6, IL-9, and IL-17 and enhancing the production of anti-inflammatory cytokines such as IL-10 [3]. This immune-modulating action suggests that vitamin D3 may help reduce airway hyperreactivity, which is a hallmark of diseases such as asthma and allergic rhinitis. In light of this, understanding the link between vitamin D3 deficiency and HRADs could offer new insights into prevention and treatment strategies.

Several epidemiological studies have suggested an inverse relationship between serum vitamin D3 levels and the incidence or severity of HRADs, particularly in children [4]. This association is especially relevant in regions with high rates of vitamin D3 deficiency, such as India, where up to 90% of children are deficient [5]. Vitamin D3 deficiency may not only predispose children to the development of HRADs but may also contribute to more frequent and severe exacerbations.

Furthermore, there is increasing interest in whether vitamin D3 supplementation could serve as an adjunct therapy to improve disease outcomes, particularly in individuals with recurrent exacerbations or poor disease control. Clinical trials have shown mixed results regarding the efficacy of vitamin D3 supplementation in reducing asthma severity and preventing respiratory infections, necessitating further research to clarify this relationship [6]. Therefore, investigating the association between vitamin D3 deficiency and HRADs could provide critical insights into disease management, particularly in populations with high deficiency rates.

This study aims to estimate vitamin D3 levels in children with HRADs in a hospital-based setting and to assess the association between vitamin D3 levels and HRADs by comparing them with the vitamin D3 levels of healthy children.

## Materials and methods

This study was a case-control investigation conducted among children aged six months to 12 years. The study was hospital-based, carried out in the Department of Paediatrics, Guru Gobindsingh Government Hospital, Jamnagar. The study was conducted between April 2023 and October 2024. The Institutional Ethics Committee of Shri Meghji Pethraj Shah Government Medical College and Guru Gobindsingh Hospital, Jamnagar, issued approval on 11/04/2023 (Ref. No.: 18/01/2023).

The sample size was calculated based on the following parameters: proportion in the control group (p0) = 0.21, expected proportion in the case group (p1) = 0.40, significance level (α) = 0.05 (two-tailed), and power (1-β) = 0.80. Using the formula for sample size determination:

"$&quot; \eta (\text{each group}) = \frac{\left( p_0 q_0 + p_1 q_1 \right) \left( z_{1-\alpha/2} + z_{1-\beta/2} \right)^2}{\left( p_1 - p_0 \right)^2} &quot;$"

where p0 = 0.21, q0 = 0.79 (1- p0 = 1-0.21=0.79), p1 = 0.40, q1 = 0.60 (1- p1 = 1-0.40=0.60), z1-α/2 = 1.96 (for 95% confidence interval), z1-β/2 = 0.84 (for 80% power). Substituting the values, the sample size was rounded to 100 for each group, resulting in a total of 200 participants (100 cases and 100 controls).

The inclusion criteria encompassed children aged six months to 12 years, with cases being children with ≥2 episodes of wheeze-associated lower respiratory tract infection (WALRI) or asthma and controls being normal healthy children taken as controls who come to immunization clinic that do not have any illness and not taking vitamin D3 supplements. The exclusion criteria included children aged below six months or above 12 years, those who are taking vitamin D3 supplements, and those who do not provide consent.

Data collection involved several steps in participant recruitment. Eligible children visiting the hospital during the study period were identified and assigned unique identification numbers. Separate lists were maintained for potential cases and controls. Simple random sampling was used to select participants. The randomization process involved a computer-generated random number list used to select participants, with separate random numbers generated for cases and controls. Selected participants were approached, and informed consent was obtained. If a participant declined to participate, the next child on the list was selected. Eligibility was verified to ensure cases met the criteria for HRADs and controls were healthy without HRAD. Replacement was done for ineligible participants.

The data collection tools included a customized, pretested proforma used to record relevant data. The proforma included demographics (age, sex, and religion), history (past history of similar complaints, family history, birth history, dietary habits, and frequency and duration of sun exposure), anthropometry (measurements such as weight, height, and body mass index (BMI)), and investigations (vitamin D3 levels, chest X-rays, and wrist X-rays).

For laboratory investigations, venous blood samples were collected under aseptic conditions. Vitamin D3 levels were measured using the electrochemiluminescence immunoassay (ECLIA) method. 

Radiological investigations included chest X-rays performed to identify hyperinflation and other respiratory abnormalities, and wrist X-rays evaluated for signs of rickets. The treatment protocol specified that participants identified as vitamin D3 deficient were treated according to the 2021 Indian Academy of Paediatrics (IAP) guidelines [7].

The statistical analysis was conducted using IBM SPSS Statistics for Windows, Version 25.0 (released 2017, IBM Corp., Armonk, NY). Statistical methods included expressing continuous variables as mean ± standard deviation and comparing them using independent t-tests. Categorical variables were presented as frequencies and percentages and compared using chi-square tests. Odds ratios (ORs) with 95% confidence intervals (CIs) were calculated to assess associations between variables and HRAD or vitamin D deficiency. Subgroup analyses were performed to examine vitamin D3 status across different age groups, nutritional statuses, and sun exposure levels using one-way ANOVA or Kruskal-Wallis tests as appropriate. A p-value <0.05 was considered statistically significant.

In preparing this manuscript, we used AI language models (ChatGPT (OpenAI, San Francisco, California) and Claude (Anthropic, San Francisco, California) solely to improve language and readability. These tools assisted with grammar and text fluency only. All scientific content, methodology, and conclusions are entirely our own work. AI tools are not listed as authors.

## Results

Statistical analysis revealed significant associations between HRADs and various factors. Younger age was linked to a higher risk of HRADs (p = 0.017). Males were about twice as likely to have HRADs as females (70% vs. 53%, p = 0.021, OR 2.06, 95% CI: 1.11-3.83). Muslim children were over twice as likely to have HRADs compared to non-Muslims (30% vs. 15%, p = 0.017, OR 2.43, 95% CI: 1.17-5.04). Vitamin D3 deficiency was strongly associated with HRAD, with deficient children being more than three times as likely to have the condition (37% vs. 15%, p = 0.001, OR 3.31, 95% CI: 1.66-6.61). However, a family history of airway disease showed no significant association (21% vs. 15%, p = 0.301, OR 1.50, 95% CI: 0.70-3.20) (Table 1).

**Table 1:** Association of variables with hyperreactive airway diseases *p-value <0.05 - significant, **p-value <0.001 - highly significant Continuous variables as mean ± standard deviation are compared using independent t-tests. Categorical variables as frequencies and percentages are compared using chi-square tests. Odds ratios (OR) with 95% confidence intervals (CI) were calculated to assess associations between variables.

Variable	Cases (N = 100)	Controls (N = 100)	Odds Ratio (95% CI)	P-value
Age (years), mean ± SD	4.1 ± 3.2	5.3 ± 3.6	-	0.017*
Sex				0.021*
Male	70 (70%)	53 (53%)	2.06 (1.11- 3.83)	-
Female	30 (30%)	47 (47%)	Reference	-
Religion				0.017*
Muslim	30 (30%)	15 (15%)	2.43 (1.17- 5.04)	-
Non-Muslim	70 (70%)	85 (85%)	Reference	-
Vitamin D3 deficiency (<20 ng/mL)	47 (47%)	18 (18%)	3.31 (1.66- 6.61)	0.001**
Family history of airway diseases	21 (21%)	15 (15%)	1.50 (0.70- 3.20)	0.301

**Figure 1:**
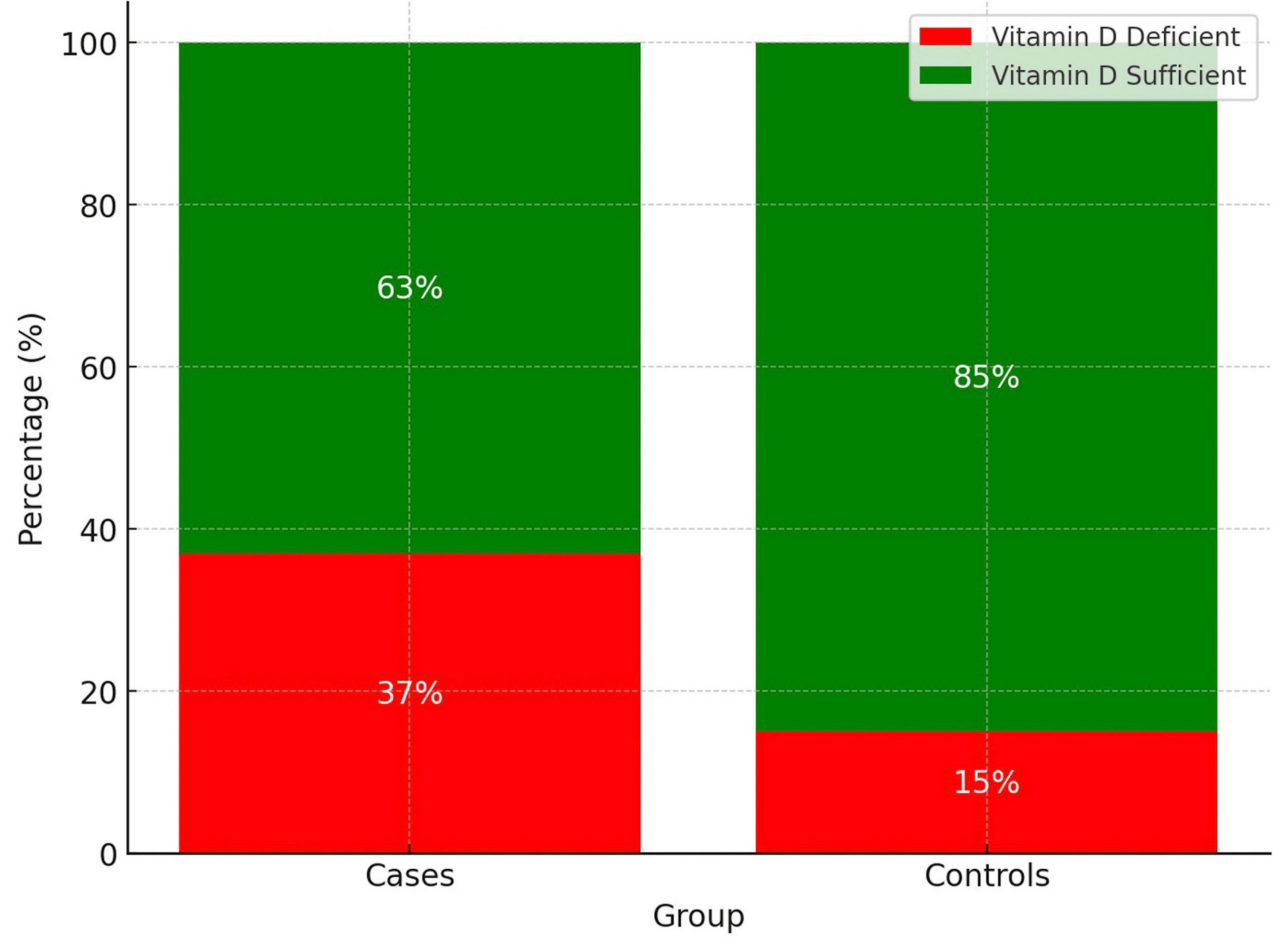
Bar chart for vitamin D status in case and control groups

The bar graph shows that 37% of participants had vitamin D3 deficiency (<20 ng/mL), highlighting a significant prevalence of deficiency in the study population. This finding indicates that nearly one-third of individuals may be at risk for health issues related to vitamin D3 deficiency, including impaired calcium absorption, bone health, immune function, and respiratory health. The results emphasize the need for increased awareness, improved nutrition, and potential supplementation strategies (Figure 1).

**Figure 2:**
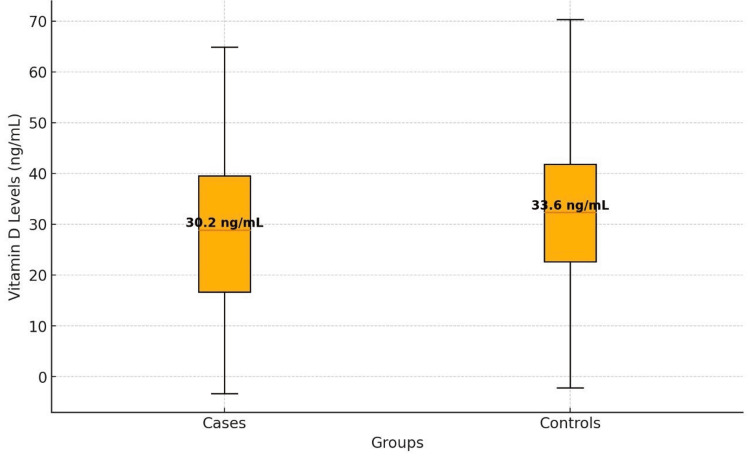
Box-Whisker plot for vitamin D levels: cases vs. controls

The box plot compares vitamin D3 levels (ng/mL) between the two groups. The case group has a median level of 30.2 ng/mL, with a box (interquartile range) spanning 17-40 ng/mL and whiskers from approximately -3 to 65 ng/mL. The control group shows a slightly higher median of 33.6 ng/mL, a narrower box from 22-42 ng/mL, and whiskers ranging from about -2 to 70 ng/mL. While the control group exhibits less variability in the middle 50%, both groups have overlapping distributions and similar overall ranges, with some extremely low values below 0 ng/mL (Figure 2).

The table summarizes the demographic characteristics of the study population by the vitamin D3 status. Among 200 participants, 65 (32.5%) were vitamin D3 deficient (<20 ng/mL), and 135 (67.5%) had sufficient levels. Vitamin D3 deficient individuals were significantly younger (2.6 ± 2.3 years) than those with sufficient levels (4.7 ± 3.5 years, p < 0.001), suggesting that younger children may be at higher risk due to factors like diet or sun exposure. Males were more prevalent in both groups (69.2% in deficient vs. 63.0% in sufficient, p = 0.38). A slightly higher percentage of Muslims were vitamin D3 deficient (30.8%) compared to sufficient (25.2%), but this was not statistically significant (p = 0.40). While age strongly correlates with vitamin D3 status, gender and religion do not appear to play significant roles (Table 2).

**Table 2:** Demographic characteristics of study participants according to vitamin D3 status *p-value <0.05 - significant, **p-value <0.001 - highly significant Continuous variables as mean ± standard deviation are compared using independent t-tests. Categorical variables as frequencies and percentages are compared using chi-square tests.

Characteristic	Vitamin D3 deficient (<20 ng/mL)	Vitamin D3 sufficient (≥20 ng/mL)	p- value
Total	65 (32.5% )	135 (67.5%)	
Age			
Mean ± SD	2.6 ± 2.3	4.7 ± 3.5	<0.001^**^
Sex			
Male	45 (69.2%)	85 (63.0%)	0.38
Female	20 (30.8% )	50 (37.0% )	
Religion			
Muslim	20 (30.8%)	34 (25.2%)	0.40
Non-Muslim	45 (69.2%)	101 (74.8%)	

Our analysis revealed significant associations between vitamin D3 status and nutritional parameters, dietary habits, and sun exposure patterns. Children over five years with vitamin D3 deficiency showed lower mean BMI (13.4 ± 1.5) compared to the sufficient group (15.1 ± 2.9, p < 0.001). Among children under five years, a higher proportion of the vitamin D3-deficient group (43.1%) exhibited moderate to severe malnutrition (Z-scores below -2 SD) compared to the sufficient group (8.1%), while 75.6% of sufficient children maintained normal nutritional status (Z-scores ≥ -1 SD) versus 27.7% in the deficient group (p < 0.001). Regarding dietary patterns, vitamin D3-sufficient children had higher consumption of butter and cheese (74.8% vs. 58.5%, p = 0.03), although egg consumption was greater in the deficient group (36.9% vs. 21.5%). Notably, 78.5% of deficient children consumed vitamin D3-rich foods less than three times weekly compared to 55.6% of sufficient children (p = 0.002), with no participants reporting consumption five times per week. Sun exposure emerged as a crucial factor, with vitamin D3 deficiency significantly associated with limited sun exposure (<15 minutes daily: 47.7% vs. 21.5%, p < 0.001). Only 7.7% of the deficient group reported more than 45 minutes of daily sun exposure, compared to 29.6% of the sufficient group, highlighting the importance of adequate sun exposure in maintaining optimal vitamin D3 levels (Table 3).

**Table 3:** Association between nutritional parameters, dietary habits, and sun exposure and vitamin D3 status *p-value <0.05 - significant, **p-value <0.001 - highly significant Subgroup analyses were performed to examine vitamin D status with nutritional status, dietary factors, and sun exposure levels using one-way analysis of variance (ANOVA).

Characteristic	Vitamin D3 deficient (<20 ng/mL)	Vitamin D3 sufficient (≥20 ng/mL)	p-value
Nutritional status			
BMI (>5 years)			
Mean ± SD	13.4 ± 1.5	15.1 ± 2.9	<0.001^**^
Z-score (<5 years)			
< -2 SD	28 (43.1%)	11 (8.1%)	<0.001^**^
-2 SD to -1 SD	19 (29.2%)	22 (16.3%)	
≥ -1 SD	18 (27.7%)	102 (75.6%)	
Dietary factors			
Predominant diet			
Butter, cheese	38 (58.5%)	101 (74.8%)	0.03^*^
Egg	24 (36.9%)	29 (21.5%)	
Frequency			
Less than three times in a week	51 (78.5%)	75 (55.6%)	0.002^*^
Three to five times in a week	14 (21.5%)	50 (37.0%)	
Five times in a week	0 (0%)	10 (7.4%)	
Sun exposure			
Less than 15 minutes	31 (47.7%)	29 (21.5%)	<0.001^**^
15 to 45 minutes	29 (44.6%)	66 (48.9%)	
More than 45 minutes	5 (7.7%)	40 (29.6%)	

Vitamin D3 deficiency is associated with significant clinical findings, including a higher prevalence of a family history of allergy (43.1% vs. 23.7%, p = 0.004), suggesting a link to allergic predisposition and immune function. Chest X-rays revealed hyperinflation in 72.3% of deficient individuals compared to 18.5% of sufficient individuals (p < 0.001), indicating a potential role in respiratory health and airway hyperreactivity. Wrist X-rays showed signs of rickets in 24.6% of deficient individuals versus 5.2% in the sufficient group (p < 0.001), underscoring vitamin D3's critical role in bone health and calcium metabolism (Table 4).

**Table 4:** Association between family history of allergy and radiological findings to vitamin D3 status *p-value <0.05 - significant, **p-value <0.001 - highly significant Categorical variables as frequencies and percentages are compared using chi-square tests.

Characteristic	Vitamin D3 deficient (<20 ng/mL)	Vitamin D3 sufficient (≥20 ng/mL)	p-value
Family history of allergy	28 (43.1%)	32 (23.7%)	0.004^*^
Chest X-ray finding			
Normal	18 (27.7%)	110 (81.5%)	<0.001^**^
Hyperinflation	47 (72.3%)	25 (18.5%)	
Wrist X-ray finding			
Normal	49 (75.4%)	128 (94.8%)	<0.001^**^
Rickets	16 (24.6%)	7 (5.2%)	

## Discussion

This study investigated the association between vitamin D3 levels and HRADs in children aged six months to 12 years. The findings highlight several significant relationships that contribute to our understanding of the role of vitamin D3 in respiratory health and related demographic, nutritional, and environmental factors.

Our study revealed a substantial prevalence of vitamin D3 deficiency (32.5%) among children with HRADs, defined as vitamin D3 levels below 20 ng/mL. This finding aligns with previous studies that have reported high rates of vitamin D3 deficiency among children in various populations [8]. While the mean vitamin D3 level in cases (30.2 ± 17.8 ng/mL) was lower than in controls (33.6 ± 14.5 ng/mL), the difference was not statistically significant (p = 0.166). However, the odds of vitamin D3 deficiency were significantly higher in cases compared to controls (OR 3.31, 95% CI 1.66-6.61, p = 0.001). These results suggest a potential link between vitamin D3 deficiency and HRAD, consistent with the hypothesis that vitamin D3 plays a role in modulating immune responses and inflammation in the airways.

Children with HRADs were significantly younger (mean age 4.1 years) than controls (5.3 years, p = 0.017), indicating a higher vulnerability to HRADs in younger children. This finding underscores the importance of early identification and intervention in this age group to mitigate the impact of HRADs.

Male sex was associated with an increased risk of HRADs (OR 2.06, 95% CI 1.11-3.83, p = 0.021). This gender disparity in respiratory diseases has been observed in previous studies and may be attributed to differences in lung development, airway size, and immune responses between sexes [9].

Children from the Muslim community were found to have a higher risk of HRADs (OR 2.43, 95% CI 1.17-5.04, p = 0.017). This association may reflect cultural or lifestyle factors, such as clothing that limits sun exposure, dietary habits, or other environmental influences, which could impact vitamin D3 status and respiratory health [10].

A family history of allergy was more common among vitamin D3-deficient individuals (43.1% vs. 23.7%, p = 0.004) and was associated with an increased risk of vitamin D3 deficiency (OR 2.44, 95% CI 1.29-4.61, p = 0.006). However, its association with HRAD was not statistically significant (OR 1.50, 95% CI 0.70-3.20, p = 0.301). These findings suggest a potential genetic or environmental link between allergic predisposition and vitamin D3 deficiency, warranting further research [11].

Table 5 compares current study findings on factors like age, sex, religion, vitamin D3 deficiency, and family history with previous study findings.

**Table 5:** Comparison of current findings with previous studies wrt to association between variables and hyperreactive airway diseases (HRADs)

Factor	Current study findings	Previous study findings	Comparison
Age	Cases were younger (mean age 4.1 years) than controls (5.3 years), p = 0.017	Huang, Kewu et al. (2019) found a higher prevalence of wheezing in children <5 years (OR: 1.65, 95% CI: 1.24-2.20) [12].	Consistent: Both studies indicate higher risk in younger children
Sex (male)	Higher risk in males (OR: 2.06, 95% CI: 1.113.83, p = 0.021)	Worldwide Asthma Prevalence Study (2022) reported higher asthma prevalence in boys (14.6%) compared to girls (11.2%) [13].	Consistent: Both studies show higher risk in males
Religion (Muslim)	Higher risk in Muslims (OR: 2.43, 95% CI: 1.17-5.04, p = 0.017)	Bener et al. (2016) found a higher prevalence of asthma in Muslim children in Qatar (19.8%) compared to general population (13.5%) [14].	Consistent: Both studies indicate higher risk in Muslim children
Vitamin D3 deficiency	Strong association (OR: 3.31, 95% CI: 1.66-6.61, p = 0.001)	Prasad et al. (2020) meta-analysis showed association between vitamin D3 deficiency and increased asthma risk (OR: 1.68, 95% CI: 1.32-2.13) [15].	Consistent but stronger association in current study
Family history	Slight increase, not significant (OR: 1.50, 95% CI: 0.70-3.20, p = 0.301)	Burke et al. (2018) found significant association between parental asthma and childhood asthma (OR: 3.0, 95% CI: 2.63.5) [16].	Inconsistent: Current study shows weaker, non-significant association

Our study demonstrated a strong association between poor nutritional status and vitamin D3 deficiency. Children with Z-scores below -2 SD had significantly higher odds of vitamin D3 deficiency (OR 11.84, 95% CI 4.71-29.74, p < 0.001). Among children older than five years, the mean BMI was significantly lower in the vitamin D3-deficient group (13.4 ± 1.5) compared to the sufficient group (15.1 ± 2.9, p < 0.001). These findings underscore the interplay between malnutrition and vitamin D3 deficiency, as previously reported [17]. This bidirectional relationship highlights the need for comprehensive nutritional interventions to address both conditions effectively.

Consumption of vitamin D-rich foods, such as butter and cheese, was associated with a lower risk of vitamin D3 deficiency (OR 0.48, 95% CI 0.25-0.90, p = 0.02). This finding emphasizes the importance of dietary sources of vitamin D3 in maintaining adequate levels, particularly in populations with limited sun exposure.

The frequency of consuming vitamin D-rich foods also played a significant role. Children with less frequent intake (≤3 times per week) had an increased risk of vitamin D3 deficiency (OR 2.91, 95% CI 1.46-5.80, p = 0.002). This highlights the need for regular dietary intake of vitamin D-rich foods to prevent deficiency.

Limited sun exposure (<15 minutes daily) significantly increased the risk of vitamin D3 deficiency (OR 5.01, 95% CI 2.27-11.05, p < 0.001). This finding reinforces the critical role of sensible sun exposure in maintaining adequate vitamin D3 levels and supports previous research linking sun exposure to respiratory health [14].

Chest X-ray hyperinflation was significantly more prevalent among vitamin D3-deficient individuals (OR 11.49, 95% CI 5.61-23.51, p < 0.001). Strikingly, 74% of cases showed hyperinflation compared to 0% in controls (p < 0.001). This supports the growing body of evidence suggesting a role for vitamin D3 in respiratory health and lung function [18].

The presence of rickets on wrist X-rays was significantly associated with vitamin D3 deficiency (OR 5.97, 95% CI 2.29-15.55, p < 0.001). Rickets were observed in 15% of cases compared to 0% in controls (p < 0.001), consistent with the well-established role of vitamin D3 in bone health [19].

Table 6 compares present study findings on vitamin D3 deficiency factors like prevalence, age, gender, nutrition, sun exposure, respiratory health, and family history of allergy with findings from previous studies.

**Table 6:** Comparison of present study results with previous studies wrt to association between variables and vitamin D3

Factor	Present study results	Previous study results
Prevalence of vitamin D3 deficiency	32.5% of the study population had levels below 20 ng/mL.	High rates of vitamin D3 deficiency were reported in asthmatic children (56%) [8].
Age and vitamin D3 deficiency	Vitamin D3-deficient individuals were younger (mean age 2.6 ± 2.3 years).	An association between younger age and vitamin D3 deficiency was observed [20]. The mean serum level of vitamin D3 was significantly lower among infants compared to children aged 12 months and more (p = 0.001).
Gender and hyperreactive airway disease	Male sex was associated with increased risk (OR 2.06, 95% CI 1.11-3.83).	Gender disparity in respiratory diseases was noted, possibly related to differences in lung development and immune responses [9,20], Males are more affected than females (28% vs. 19%).
Nutritional status and vitamin D3 deficiency	A strong association between poor nutritional status and vitamin D3 deficiency (OR 11.84, 95% CI 4.71-29.74 for Z-scores below -2 SD).	The complex interplay between overall nutrition and vitamin D3 status was reported [17] (OR = 2·0; 95 % CI 1·2, 3·4).
Sun exposure and vitamin D3 deficiency	Limited exposure (<15 minutes daily) increased the risk of vitamin D3 deficiency (OR 5.01, 95% CI 2.27-11.05).	The importance of sensible sun exposure in maintaining adequate vitamin D3 levels was reported in numerous studies [18] (OR = 1.73; p = 0.003).
Vitamin D3 and respiratory health	A strong association exists between vitamin D3 deficiency and chest X-ray hyperinflation (OR 11.49, 95% CI 5.61-23.51).	A growing body of evidence suggests a role for vitamin D3 in respiratory health and lung function [19].
Family history of allergy	More common in vitamin D3-deficient individuals (43.1% vs. 23.7%, p = 0.004).	A study by Bener et al. found that children with a family history of allergy who were also deficient in vitamin D3 were significantly more likely to develop asthma (OR = 4.6, p < 0.001) compared to those with sufficient vitamin D3 levels and no family history [11].

While our study provides valuable insights, it has some limitations. The cross-sectional design precludes establishing causality between vitamin D3 deficiency and HRADs. In addition, we did not account for all potential confounding factors, such as genetic predisposition or environmental pollutants. The sample size, while adequate for many analyses, may have limited the power to detect some associations. One study found that the Roche Elecsys Vitamin D3 (25OH) ECLIA method underestimated vitamin D3 levels and was removed from use by Roche Diagnostics.

Longitudinal studies are essential to establish the causal relationship between vitamin D3 status and HRADs. Intervention trials should evaluate whether vitamin D3 supplementation improves clinical outcomes in children with HRADs. In addition, comprehensive research into the roles of diet, sun exposure, and cultural factors influencing vitamin D3 levels and respiratory health is warranted.

Screening for vitamin D3 deficiency should be considered in children with HRADs, particularly those with poor nutritional status, a family history of allergy, or limited sun exposure. Prophylactic vitamin D3 supplementation may be beneficial for these high-risk groups to mitigate potential adverse outcomes. This targeted approach could improve the management and prevention of HRADs while addressing vitamin D3 deficiency in vulnerable populations.

## Conclusions

Our study demonstrates a significant association between vitamin D3 deficiency and HRADs in children, despite the fact that the cutoff for vitamin D3 deficiency has been lowered to 20 ng/mL. The complex interplay between age, sex, religion, nutritional status, dietary factors, sun exposure, and clinical manifestations highlights the multifaceted role of vitamin D3 in pediatric respiratory health. These findings underscore the importance of monitoring vitamin D3 status in children, particularly those with respiratory symptoms, and suggest that interventions to improve vitamin D3 status may have potential benefits in managing HRADs.
